# Tributes to Medical Students

**Published:** 1887-03

**Authors:** 


### TRIBUTES TO MEDICAL STUDENTS.

KINTZ.—The untimely death, at 7 o'clock p. m., on the 5th of February, 1887, of Mr. J. E. KINTZ, from Summerset, Ohio, has cast a gloom over the Southern Medical College, with which his association as a member of the Senior Class during the present term has endeared him to the Faculty and Students.

Having left his kindred and friends in search of a more genial climate, on account of a supposed tendency to pulmonary disease, he had realized all the benefits anticipated in the improvement of his general health, and had found in his new relations a compensation for the tender ties which had been severed at home, but most unexpectedly fell a victim to an overpowering congestive attack, which resisted the skill of assiduous and capable medical attendants.

The Faculty of the Southern Medical College realize the great loss sustained in his death, and feel deeply impressed with the conviction that he would have proved an ornament to the medical profession in which he was shortly to have entered amongst the foremost of his class. We, therefore, give this expression of appreciation for his worth and our profound sympathy in the bereavement of his family :

Resolved, That the exercises of this Institution be suspended during the day in consideration of respect to our friend and student.

Resolved, That the Faculty and entire body of Students accompany the body of our deceased friend to the depot when it may be removed.

Resolved, That copies of this be furnished for publication in our daily papers, and sent to his parents, to commemorate the high estimate placed upon his character.

J. McF. GASTON, M. D.,
Professor of Surgery.
W. D. BIZZELL, M. D.,
Professor of Practice.
G. G. ROY, M. D.,
Professor of Materia Medica and Therapeutics.

---

STILES.—At a meeting of the Faculty, held February 5, 1887, the following preamble and resolutions were adopted, unanimously :

The Professors and Students of the Southern Medical College regard the death of Mr. C. STILES, a member of the present Class, a very sad dispensation, and the Faculty hereby express their deep sympathy with his family in their sorrow.

A young man of unblemished character, and of great devotion to his medical pursuits, Mr. STILES bid fair to attain distinction in his profession ; and, as his instructors, we deeply regret that he should have been cut off so early in his career of usefulness. It is therefore

Resolved, That the Southern Medical College has sustained an irreparable loss in his untimely death, and that the Faculty share in the bereavement with his fellow-students.

Resolved, That, as a mark of consideration, this action be recorded upon the Register of this Institution.

Resolved, That a copy of this paper be forwarded to his parents, as an indication of our regard and condolence in their affliction.

Being appointed as a Committee for this purpose, we hereunto affix our names.

J. McF. GASTON, M. D.,
Professor of Surgery.
A. G. HOBBS, M. D.,
Professor Eye, Ear and Throat Diseases.
WM. PERRIN NICOLSON, M. D.,
Professor of Anatomy.

